# A Quantum-Based Microwave Magnetic Field Sensor

**DOI:** 10.3390/s18103288

**Published:** 2018-09-30

**Authors:** Hao Shi, Jie Ma, Xiaofeng Li, Jie Liu, Chao Li, Shougang Zhang

**Affiliations:** 1 Key Laboratory of Time and Frequency Standards, National Time Server Center, Chinese Academy of Sciences, Xi’an 710600, China; 18392856723@163.com (H.S.); lixiaofeng@ntsc.ac.cn (X.L.); liujie@ntsc.ac.cn (J.L.); lichao@ntsc.ac.cn (C.L.); szhang@ntsc.ac.cn (S.Z.); 2 School of Astronomy and Space Science, University of Chinese Academy of Sciences, Beijing 100049, China

**Keywords:** atomic measurement, microwave magnetic field, atomic candle, laser

## Abstract

In this paper, a quantum-based method for measuring the microwave magnetic field in free space is presented by exploring atomic Rabi resonance in the clock transition of ^133^Cs. A compact cesium glass cell serving as the microwave magnetic field sensing head was used to measure the spatial distribution of microwave radiation from an open-ended waveguide antenna. The measured microwave magnetic field was not restricted by other microwave devices. The longitudinal distribution of the magnetic field was measured. The experimental results measured by the sensor were in agreement with the simulation. In addition, a slightly electromagnetic perturbation caused by the glass cell was investigated through simulation calculations.

## 1. Introduction

In many production areas, building measurement of the electromagnetic field in the radio-frequency (RF) range in a free space is either expected or required. At present, the main methods of measuring RF electromagnetic field strength are the calorimetric method [1,2,3] and the technique of peak demodulation [4]. However, the field strength cannot be measured directly by either method, and the measurement principle of these methods is conversion of the field strength to measurable electrical signals. In addition, the calorimetric method is greatly affected by temperature and the performance of the technique of peak demodulation will worsen when the field strength is reduced. Moreover, with these methods, a detector that consists of metallic parts can perturb the electromagnetic field distribution and thus the detector must be calibrated when absolute measurement results are needed.

Recently, quantum-based RF field measurements have attracted attention for their simple and non-metallic setup [5,6,7,8,9,10,11,12,13,14,15,16,17,18,19,20,21,22,23,24,25,26,27,28,29,30,31,32]. Alkali atoms in Rydberg states have been utilized to measure microwave electric fields [14,15,16,17,18,19,20,21,22]. Specifically, the Rabi frequency associated with atomic transition is proportional to the electromagnetic field strength. Moreover, the Rabi frequency can be conveniently measured by a field-amplitude stabilization technique, based on the second harmonic oscillation of the atomic population, when a phase modulation is added to the RF electromagnetic field. For ease of reference, and by analogy to the atomic clock, we refer to the Rabi resonance, field-amplitude stabilization process as an ‘‘atomic candle’’ [11,12]. The measurement of absolute electromagnetic field strength can be realized and the measurement process is substantially free from interference because the proportionality constant involved in the quantum-based method is determined only from fundamental physical constants and atomic quantum based constants. Furthermore, the quantum-based electromagnetic field measurement method is expected to be of higher accuracy and quicker than the present method. Compared with the metallic sensing head, the perturbation for the targeted electromagnetic field caused by glass sensing head can be reduced [21].

In this paper, we report on a new sensor for measuring the spatial distribution of the magnetic field strength in a perpendicular direction to the microwave ejection plane of a waveguide. The RF electromagnetic field was radiated from the open-end waveguide. The magnetic field strength was read by the atomic candle spectrum, observed by a fast Fourier transform (FFT) analyzer. In addition, the measured results were compared with simulating calculation results to verify the function of the sensor. Electromagnetic disturbance caused by the glass cell was also analyzed using simulation calculations. The method using a Cs glass cell as the antenna to detect microwave magnetic fields is generally so simple. This sensor can be applied in many fields, such as integrated circuit manufacturing, military, communications, and medical rescue.

## 2. Principle

In our experiment, the quantum-based method refers to using an atomic candle to measure the microwave magnetic field in free space. Theoretically, atoms can be pumped from the lower state to the upper state and return, when it interacts with an electromagnetic field at the frequency that corresponds to the energy difference between these two levels. According to Reference [11], when an electromagnetic field with phase modulation at a frequency of *ω_m_* and a modulation amplitude of *θ* interacts with a two-level atom, the probability of finding the atoms in the upper-state, *P*_0_, has an oscillating component at a modulation frequency of *ω_m_* and harmonic frequency of 2*ω_m_*. According to a calculation based on density-matrix evolutions, *P*_0_ can be theoretically written as:

(1) $$P_{0}=P_{\alpha }(t)+P_{\beta }(t)$$

where *P_α_*(*t*) and *P_β_*(*t*) are the first- and second-harmonic probability oscillations and *t* is a time variable. *P_α_*(*t*) is written as:

*P_α_*(*t*) = ∆*P_α_sin*(*ω_m_t* + *ϕ*_1_) (2)

where *ϕ*_1_ is the phase, ∆ is the average field-atom detuning, *P_α_* is the amplitude of first harmonic probability oscillation, which is written as:

(3) $$P_{\alpha }\propto \frac{\theta \omega_{m}\Omega^{2}}{\gamma_{2}\sqrt{{\left(\Omega^{2}-\omega_{m}^{2}\right)}^{2}+\gamma_{1}^{2}\omega_{m}^{2}}}$$

where Ω is the Rabi frequency, the oscillation frequency of the population of atomic states, *γ*_1_ and *γ*_2_ are the longitudinal and transverse relaxation rates. *P_α_* is maximal when Ω = *ω_m_*.

However, *P_α_*(*t*) is equal to 0 when the electromagnetic field is exactly on-resonance (∆ = 0), and *P*_0_ is written as:

*P*_0_ = *P_β_*(*t*) = *P_β_ sin*(2*ω_m_t + ϕ*_2_) (4)

where *ϕ*_2_ is the phase, and *P_β_* is the amplitude of second harmonic probability oscillation, which is written as:

(5) $$P_{\beta }\propto \frac{\theta^{2}\omega_{m}\Omega^{2}}{\sqrt{{\left(\Omega^{2}-4\omega_{m}^{2}\right)}^{2}+4\gamma_{1}^{2}\omega_{m}^{2}}}$$

Obviously, *P_β_* is maximal when Ω = 2*ω_m_*. In other words, the probability of finding the atoms in the upper-state displays a resonant enhancement when Ω takes on specific values. Note that laser absorption is proportional to the existence probability on the upper-state when the resonated laser interacted with the two-level atom. Since the *P_α_* and *P_β_* may be experimentally distinguished, the resonant behaviors of the first- and second-harmonic probability oscillations amplitudes were termed as the *α* and *β* Rabi resonances, respectively. According to quantum theory, Ω is written as:

(6) $$\Omega =\frac{g_{J}\mu_{0}\mu_{B}\langle F,m_{F}|J|F+1,m_{F}\rangle }{\hbar }H$$

where *H* is the magnetic field strength, *ħ* the reduced Planck’s constant, *g_J_* the Landé g-factor, *μ*_0_ the permeability, *μ_B_* the Bohr magneton, and $\langle F,m_{F}|J|F+1,m_{F}\rangle$ the matrix element, *J* the component of the electron angular momentum. The objective of this experiment is to determine *H* by measuring the number of atoms in the *F* = 3 and *F* = 4 states after exposure to resonant microwaves [13]. Thus, Ω can be adjusted by *H*, and it gives the maximum *P*_0_ when the magnetic field strength is adjusted to specific values. Thus, the quantum-based measurement of the microwave magnetic field strength can be realized. Moreover, as Equation (6) shows, the proportional relation between Ω and *H* is determined ultimately by the fundamental physical constants. Theoretically, the experiment is not influenced by outside conditions.

**Figure 1:**
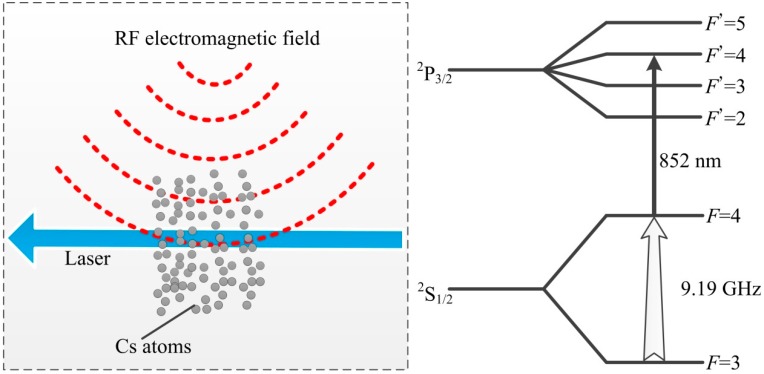
The left side is diagram of the interaction between laser and Cs atoms when the RF electromagnetic field is present. And the right side is diagram of energy level transition made in the interaction.

As shown in Figure 1, a Cs atom can be transported from *F* = 3 of the ground state to *F* = 4 of the ground state by the RF electromagnetic field at a clock frequency of 9.19263177 GHz. The population of *F* = 4 can be increased. Then, it can be pumped from *F* = 4 of the ground state to *F*′ = 4 of the excited state by the laser locked in the *F* = 4 → *F*′ = 4 transition of the Cs D_2_ line [33]. In this process, the laser is absorbed by Cs atoms, and the laser absorption can be increased because of the increase of the Cs atom population of *F* = 4 when the RF electromagnetic field is present. The laser absorption have a maximum value when the population of *F* = 4 reach to maximum. The laser absorption has an oscillating component corresponding to harmonics oscillation of the Cs atom population of *F* = 4, when the phase of the RF electromagnetic field is modulated. 

The interaction between an atom and a RF electromagnetic field is very weak and cannot be observed directly only with a RF electromagnetic field. In this experiment, the interaction between Cs atoms and a phase-modulated RF electromagnetic field was observed by measuring the intensity of the transmitted laser resonated by Cs atoms. The double resonance experiment [34] was processed. As we know, *H* is proportional to the square root of the RF electromagnetic field strength ($\sqrt{P}$). According to the above-mentioned quantum-based theory, the atomic candle signal was obtained when the values of the laser absorption varied with $\sqrt{P}$ were recorded. The amplitude of oscillating laser absorption is maximal when the phase-modulated RF electromagnetic field strength is adjusted to specific values.

## 3. Experiment and Results

In our experiment, Cs vapor enclosed in a cylindrical glass cell was used to detect microwaves. N_2_ buffer gas at a pressure of 40 torr was enclosed in the glass cell to slow the collision of Cs atoms with the glass walls, which can destroy the effect of optical pumping. Glass cell was a cylinder and column basal plane was the base of a cylinder. The diameter of the column basal plane of the cylindrical glass cell was 20 mm, its height was 10 mm, and its wall thickness was 0.5 mm. An open-ended waveguide was used as an emitter to send out microwaves. The dimensions of the waveguide were 22.86 mm × 10.16 mm × 17 mm, which allows the transmission of microwaves at nearly 9.19 GHz. A side of the waveguide was opening and its dimensions were 22.86 mm × 10.16 mm. This side was defined as ejection plane of the waveguide. 

**Figure 2:**
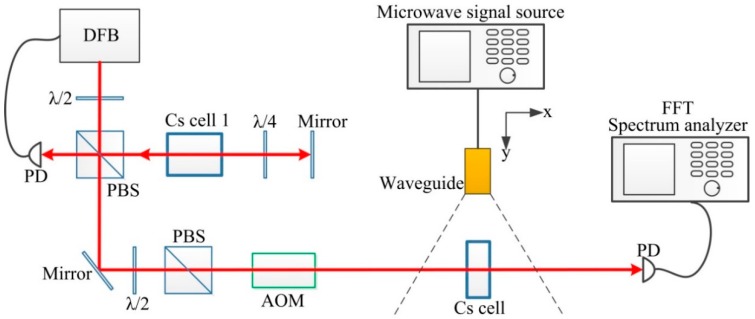
Experimental setup of quantum-based method. Cs cell 1 was used to generate saturation absorption spectroscopy. Cs cell was used to measure microwave magnetic field. The frequency of the laser was locked by the saturation absorption system.

As shown in Figure 2, the collimated laser with a wavelength of 852 nm and linewidth of 2 MHz was emitted from a commercial distributed-feedback (DFB) diode laser. A combination of a λ/2 plate and a polarizing beam splitter (PBS) was used to adjust the laser intensity. The laser was locked in the *F* = 4 → *F*′ = 5 transition of the Cs D_2_ line by Cs saturation absorption spectroscopy. And, the frequency of the laser was shifted 250 MHz by an acoustic optical modulator. The laser was stabilized to the *F* = 4 → *F*′ = 4 transition of the Cs D_2_ line. Cs atoms enclosed in the glass cell were pumped from *F* = 4 of the ground state to *F*′ = 4 of the excited state by the laser, and the transmitted laser was detected by a photodiode (PD). The detected signal was analyzed by the FFT analyzer. The diameter of the laser spot was 3 mm and the laser intensity was 85 μW/cm^2^. The direction of laser propagation was perpendicular to the column basal plane of the cylindrical Cs glass cell.

The microwave at the frequency of nearly 9.19 GHz was generated by the microwave signal source. Cs atoms were irradiated by microwaves, radiated from the open-ended waveguide, and transported from *F* = 3 of the ground state to *F* = 4 of the ground state. The Cs glass cell was put in a position towards the ejection plane of the waveguide. The centers of the waveguide and the Cs cell were placed in the same horizontal plane. The normal vector of the column basal plane of the cylindrical Cs glass cell was parallel to the microwave ejection plane of the waveguide. The distance between the ejection plane of the waveguide and the center of the Cs glass cell was defined as the measurement distance. The y-axis was perpendicular to the ejection plane of the waveguide.

$\sqrt{P}$ was adjusted by the microwave signal source and a sinusoidal phase modulation at a frequency of *ω_m_*/2π was added to the microwave by the microwave signal source. The phase modulation index of *θ* was set 2.3 rad. The harmonic oscillation was induced on the transmitted laser when the laser and the phase-modulated microwave simultaneously interacted with Cs atoms. The first- and the second-harmonic oscillations were all induced on the transmitted laser when the microwave was near-resonance, and two signal peaks at frequencies of *ω_m_*/2π and 2(*ω_m_*/2π) were read out on the FFT analyzer. Moreover, only the second harmonic oscillation was induced when the microwave was exactly on-resonance, and only one signal peak at the frequency of 2(*ω_m_*/2π) was observed in the FFT analyzer. The resonance frequency of the microwave was confirmed by the above-mentioned process, and the resonance frequency was slightly shifted about 33.8 kHz from 9.19263177 GHz due to the presence of N_2_ buffer gas. It was consistent with theoretical calculating value of 33.4 kHz [35,36]. The amplitude of the signal peaks at the frequency of 2(*ω_m_*/2π) corresponded to harmonic oscillation of the Cs atom population of *F* = 4. The amplitude of the signal peaks at each $\sqrt{P}$ was recorded; then, the atomic candle signal was obtained as a function of $\sqrt{P}$.

**Figure 3:**
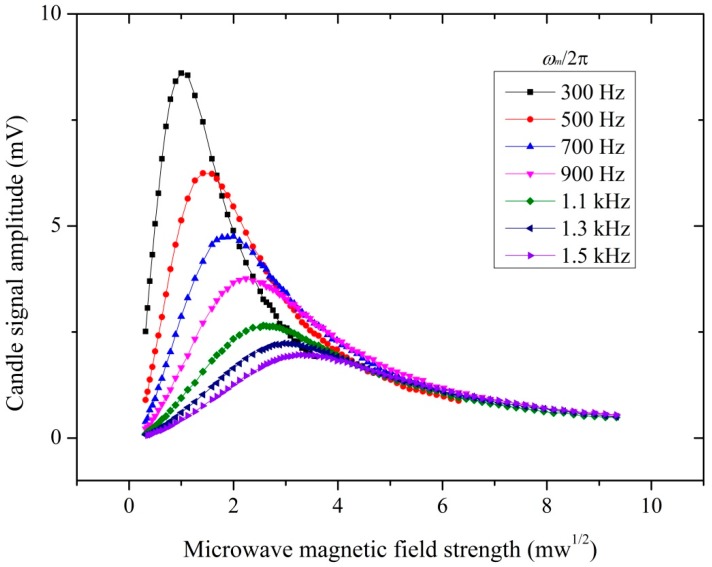
Atomic candle signal at phase-modulating frequencies of *ω_m_*/2π (300 Hz, 500 Hz, 700 Hz, 900 Hz, 1.1 kHz, 1.3 kHz, and 1.5 kHz). The detected signal was analyzed by the FFT analyzer. Vertical axis values of the plots were the peak value of the laser absorption signal observed by FFT analyzer. Horizontal axis values were observed in microwave signal source. These curves were measured at different microwave phase-modulating frequency. What we focus are the horizontal axis values of the atomic candle signal peak. The signal peak values were increased with the microwave phase-modulating frequency. The diameter of the laser spot was 3 mm and the laser intensity was 85 μW/cm^2^. The phase modulation index of *θ* was set 2.3 rad. The measurement distance was 20 mm.

**Figure 4:**
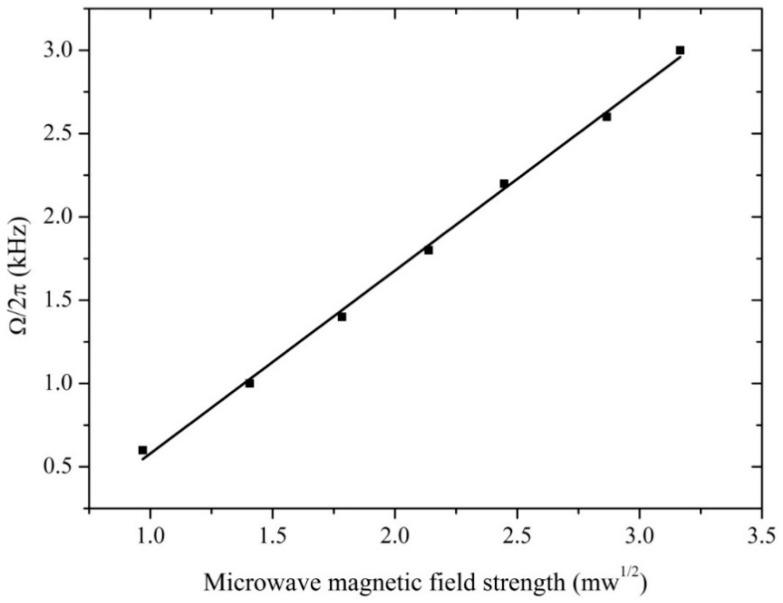
Dependence of Rabi frequency on microwave magnetic field strength. Plots were obtained from Figure 3. Horizontal axis values of plots were abscissa values of the atomic candle signal peak at different microwave phase-modulating frequencies of *ω_m_*/2π (300 Hz, 500 Hz, 700 Hz, 900 Hz, 1.1 kHz, 1.3 kHz, and 1.5 kHz). Vertical axis values of the plots were the corresponding Rabi frequencies of 2(*ω_m_*/2π) (600 Hz, 1 kHz, 1.4 kHz, 1.8 kHz, 2.2 kHz, 2.6 kHz, and 3.0 kHz). The straight line was fitted by the measured plots. The Rabi frequency is proportional to the microwave field strength, which is expected from Equation (6). The diameter of the laser spot was 3 mm and the laser intensity was 85 μW/cm^2^. The phase modulation index of *θ* was set 2.3 rad.

Figure 3 shows the atomic candle signal at the phase-modulating frequencies of *ω_m_*/2π (300 Hz, 500 Hz, 700 Hz, 900 Hz, 1.1 kHz, 1.3 kHz, and 1.5 kHz). Theoretically, the corresponding Rabi frequencies were 2(*ω_m_*/2π) (600 Hz, 1 kHz, 1.4 kHz, 1.8 kHz, 2.2 kHz, 2.6 kHz, and 3.0 kHz) when each atomic candle signal was maximal. The microwave magnetic field strengths were evaluated from the $\sqrt{P}$ set by microwave signal source and the microwave magnetic field strength was proportional to $\sqrt{P}$. As shown in Figure 3, the peak of the atomic candle signal theoretically corresponds to Ω = 2*ω_m_*, and the horizontal axis value of the peak of the atomic candle signal increased with *ω_m_*/2π. Figure 4 shows the dependence of inferred Rabi frequency on microwave magnetic field strength. The Rabi frequency was proportional to the microwave magnetic field strength, a result in accord with (4). This allows us to use the quantum-based method to measure the magnetic field strength, and then realize the microwave magnetic field sensor.

**Figure 5:**
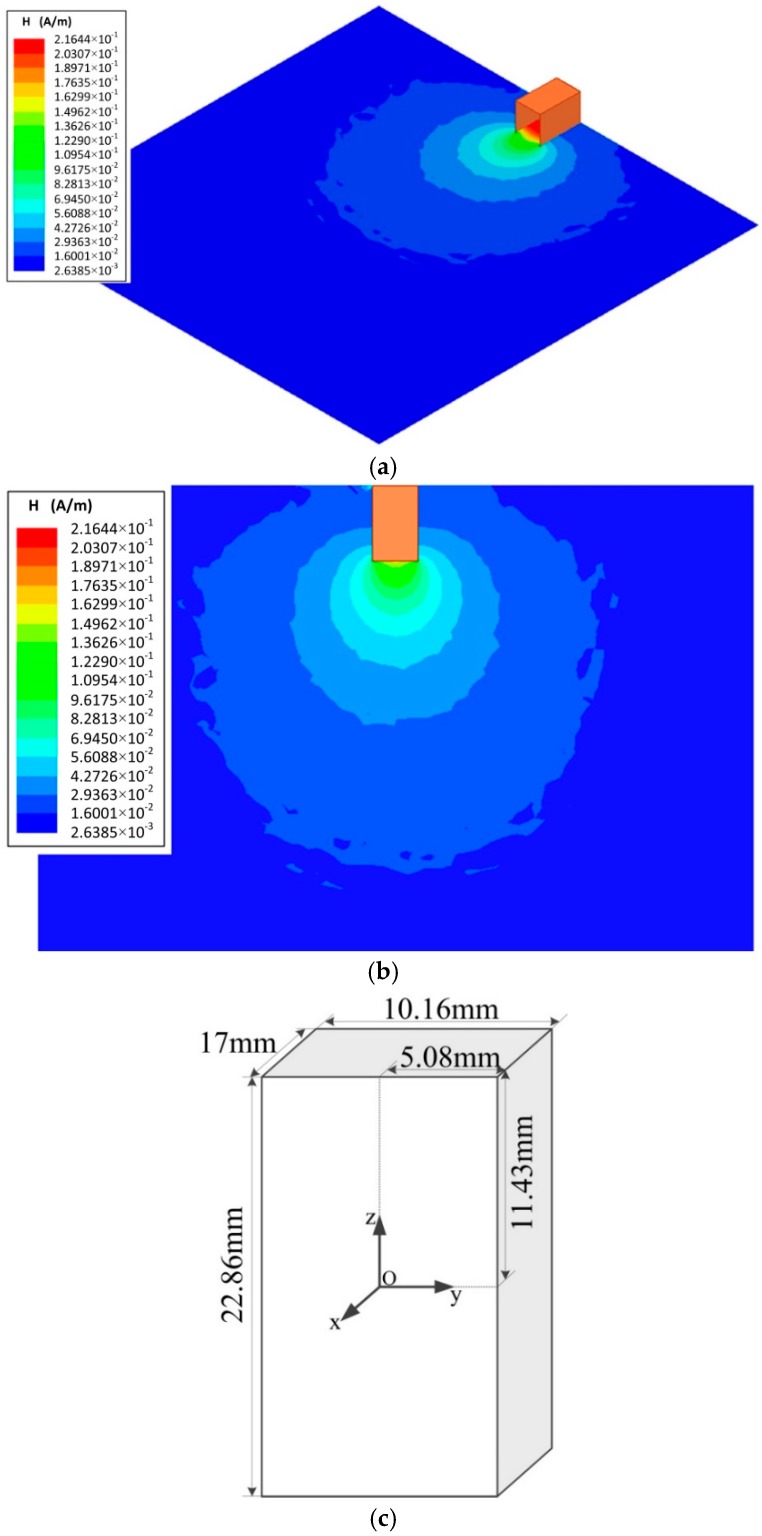
Simulation result of distribution of microwave magnetic field strength. (**a**) Three-dimensional diagram of magnetic field distribution. (**b**) Two-dimensional diagram of magnetic field distribution. (**c**) Three-dimensional diagram of the open-ended waveguide. Observation plane of the simulated distribution of microwave magnetic field strength was in the x-y plane. The size of the observation plane was 160 mm × 160 mm. A open-ended waveguide was placed at the upside. Electromagnetic waves were radiated to the down at 9.19 GHz. The dimensions of the open-ended waveguide were 22.86 mm × 10.16 mm × 17 mm.

**Figure 6:**
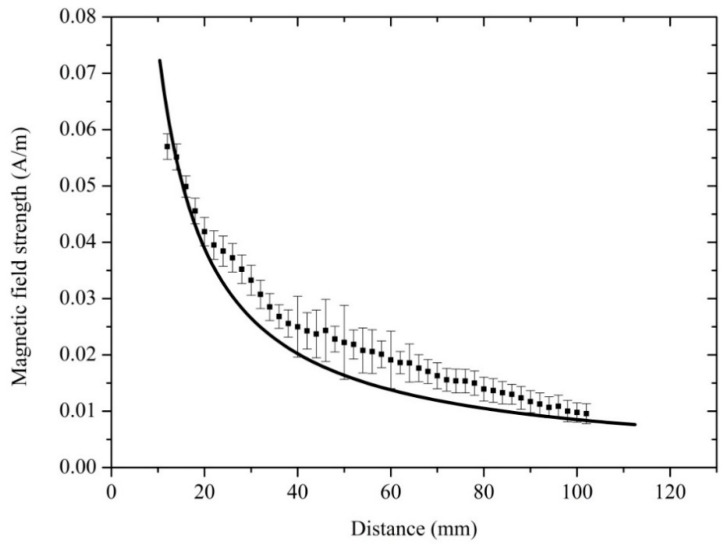
The distribution of microwave magnetic field strength along the direction of o-x (showed in Figure 5c). The square solid spot represents the measured magnetic field strength, and the solid line represents the simulated field strength. The simulated data were extracted from the results showed in Figure 5b. The magnetic field strengths were measured by changing the position of the Cs glass cell along the direction of o-x. The measurement distance was changed from 12 to 102 mm and *ω_m_*/2π *=* 500 Hz. The variation trend of the measured results was roughly identical to the simulated results. The measured results are a little more than the simulated results at every measurement points. It is because the glass cell had a resonance effect on the microwave magnetic field when the Cs glass cell was inserted. The error bars on square solid spot represents the measurement uncertainty.

The validity of the experiment was verified by the results showed in Figure 4. The spatial distribution of a magnetic field strength was measured using the quantum-based method. The measurement distance was changed from 12 to 102 mm by changing the position of the Cs glass cell and its moving direction was parallel to o-x (as the Figure 5c shows). The center of the Cs glass cell was regarded as the measurement point and the location of the measurement point was varied with measurement distance. Theoretically, with the increasing of the measurement distance, the microwave magnetic field strength at the measurement point decreases. What we focus was the spatial distribution trend of a magnetic field strength. The power of the microwave generated by the microwave signal source was adjusted to obtain the atomic candle signal when the Cs glass cell was placed at a measurement points. The microwave magnetic field strength at the atomic candle signal peak was measured when *ω_m_*/2π = 500 Hz. According to the above content, square root of the microwave powers read from the microwave signal source constituted horizontal axis of the atomic candle signal. According to the Equation (6), because the *ω_m_* was invariable, the measured microwave magnetic field strengths at the atomic candle signal peak were equal to each other when the Cs glass cell was placed at different measurement points. But, the horizontal axis value corresponding to the peak of the atomic candle signal were different when the Cs glass cell was placed at different measurement points. The power of the microwave generated by the microwave signal source should be increased to obtain the atomic candle signal peak when the measurement distance was increased. Actually, the proportional relation between the square root of the microwave powers read from the microwave signal source and the microwave magnetic field strength at the measurement point was not changed with the power of microwave generated by the microwave signal source. The distribution trend of the microwave magnetic field strength in the direction of o-x (as the Figure 5c shows) can be expressed by a series of measured values. The measured value of the 12 mm was calculated out according to the Equation (6) when *ω_m_*/2π = 500 Hz, and other measured values was expressed by the products of the measured value of the 12 mm and the ratio of two horizontal axis values. One was the horizontal axis value corresponding to the peak of the measured atomic candle signal when the measurement distance was set to 12 mm, and the other was the horizontal axis value corresponding to the peak of the measured atomic candle signal when the measurement distance was set to other values. The measured value vs. measurement distance was plotted. The distribution of the magnetic field strength in the perpendicular direction to the microwave ejection plane of the waveguide was obtained and is shown in Figure 6. 

To verify the reliability of the measurement results, the distribution of the microwave magnetic field strength was simulated by the Ansoft high frequency structure simulator (HFSS) (ANSYS, Pittsburgh, PA, USA). The square solid spot in Figure 6 represents the magnetic field strength measured by the quantum-based sensor, and the solid line represents the field strength simulated by the Ansoft HFSS. The simulation results are shown in Figure 5. The frequency of the simulation was 9.19 GHz. The microwave radiated from the open-ended waveguide with the dimensions of 22.86 mm × 10.16 mm × 17 mm. As shown in Figure 6b, the size of the two-dimensional diagram of magnetic field distribution was 160 mm × 160 mm.

The distribution of magnetic field strength measured by the quantum-based sensor and simulated by the Ansoft HFSS is plotted in Figure 6. The magnetic field strength decreased with increasing measurement distance. As Figure 6 shows, the measured and simulated magnetic field distributions are in agreement.

## 4. Discussion

The electromagnetic field perturbation caused by the Cs glass cell used in the quantum-based sensor is lower than that caused by a metal-antenna used in calorimeters. However, the perturbation introduced by the Cs glass cell remained in the measurement. We investigated the electromagnetic field perturbation by the simulation calculations when the glass cell was inserted. The distribution of microwave magnetic field strength was simulated when we set the diameter of the column basal plane of glass cell to 20 mm, its height to 10 mm, and its thickness to 0.5 mm. The normal vector of the column basal plane of the cylindrical Cs glass cell was parallel to the microwave ejection plane of the waveguide. The simulation frequency was 9.19 GHz.

**Figure 7:**
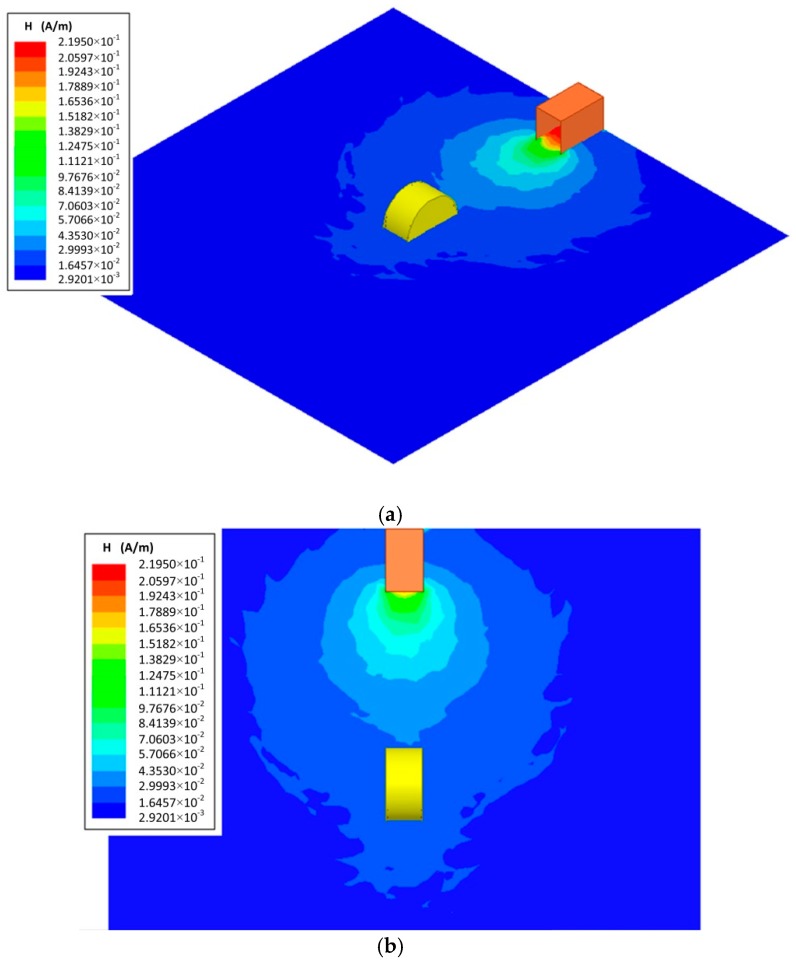
Simulation result of distribution of microwave magnetic field strength when glass cell was inserted. (**a**) Three-dimensional diagram of magnetic field distribution. (**b**) Two-dimensional diagram of magnetic field distribution. The yellow cylinder on the distribution map represents the glass cell. The size of the observation plane was 160 mm × 160 mm. A waveguide was placed at the upside. Electromagnetic waves were radiated to the down at 9.19 GHz. The dimensions of the open-ended waveguide were 22.86 mm × 10.16 mm × 17 mm. We set the diameter of the column basal plane of glass cell to 20 mm, its height to 10 mm, and its thickness to 0.5 mm. The measurement distance was 52 mm. As shown in Figure 5c, observation plane of the distribution of microwave magnetic field strength was in the x-y plane (as the Figure 5c shows).

As shown in Figure 5 and Figure 7, the microwave magnetic field strength inside the glass cell is higher than that in same place without the glass cell. The distribution of microwave magnetic field strength was slightly perturbed by the glass cell. It is expected that a resonance inside the glass cell occurs as the size of the glass cell matched that of the rectangular waveguide in the experiment. 

**Figure 8:**
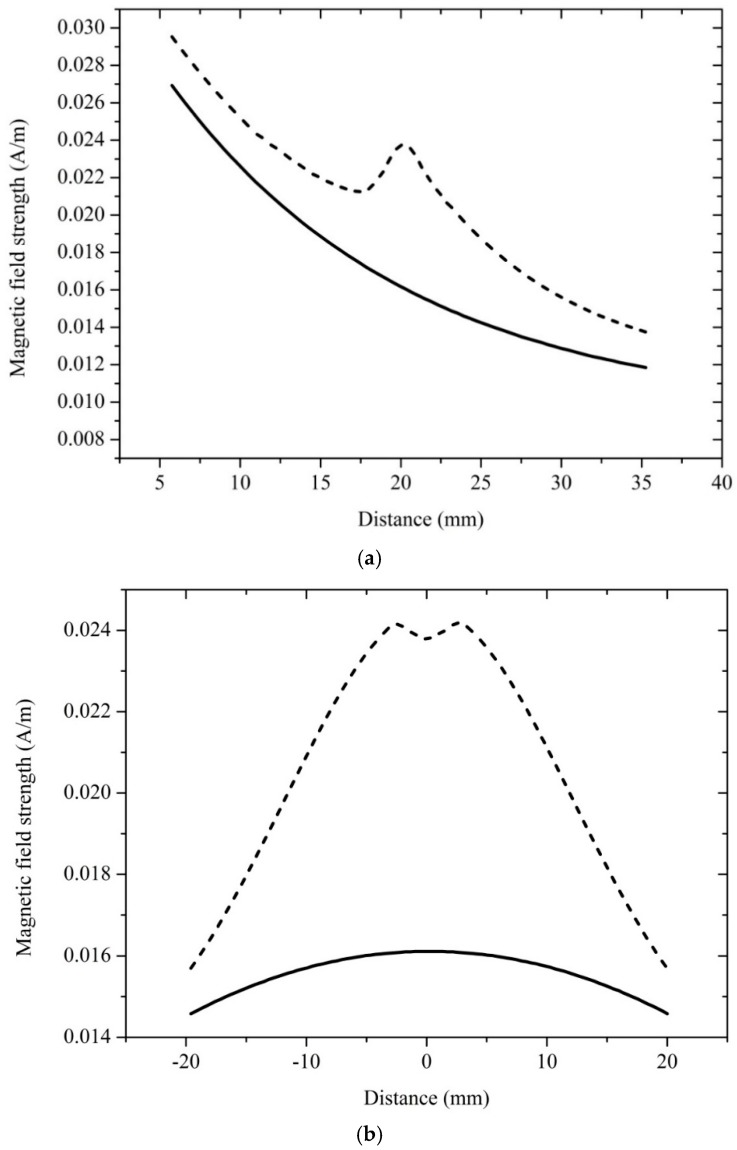
Simulation results of distribution of microwave magnetic field strength. The dash line represents the results with glass cell, and the solid line represents the results in free space. The simulation model was designed as the Figure 7a shows. Electromagnetic waves were radiated to the down at 9.19 GHz. The dimensions of the open-ended waveguide were 22.86 mm × 10.16 mm × 17 mm. We set the diameter of the column basal plane of glass cell to 20 mm, its height to 10 mm, and its thickness to 0.5 mm. The measurement distance was 52 mm. (**a**) The distribution of magnetic field strength in the direction of o-x (as the Figure 5c shows). Position 20 of horizontal axis was defined as the center of the glass cell at a measurement distance of 52 mm. The diameter of the column basal plane of the glass cell was 20 mm (10 to 30 mm). In the scope of 10 to 30 mm, magnetic field strength in the glass cell is larger than magnetic field strength in the free space. (**b**) Field strength in perpendicular direction to o-x (as the Figure 5c shows). Position 0 of horizontal axis was defined as the center of the glass cell at a measurement distance of 52 mm. The height of the glass cell was 10 mm (−5 to 5 mm). In the scope of −5 to 5 mm, magnetic field strength in the glass cell is larger than magnetic field strength in the free space.

For a more detailed discussion of the electromagnetic field perturbation caused by the Cs glass cell, the simulation results of distribution of microwave magnetic field strength in the perpendicular and parallel direction to the microwave ejection plane of the waveguide are shown in Figure 8, which corresponds to Figure 5 and Figure 7. The comparison of the simulated distribution of the magnetic field with glass cell and the magnetic field in the free space are shown in Figure 8. 

As shown in Figure 8a, the distributions of the microwave magnetic field strength are in the perpendicular direction to the microwave ejection plane of the waveguide. Position 20 was defined as the center of the glass cell at a distance of 52 mm from the microwave ejection plane of the waveguide. The diameter of the column basal plane of the glass cell was 20 mm (10 to 30 mm). As shown in Figure 8b, the distributions of microwave magnetic field strength are in the parallel direction to the microwave ejection plane of the waveguide. Position 0 was defined as the center of the glass cell at a distance of 52 mm from the microwave ejection plane of the waveguide. The height of the glass cell was 10 mm (−5 to 5 mm). As the Figure 8 shows, the glass cell had a resonance effect on the microwave magnetic field. Moreover, the microwave magnetic field strength at the center of the glass cell was visibly enhanced. The size of the glass cell is the principal factor that effects the distribution of the microwave magnetic field strength. The radiation properties of the waveguide may have been modified by the glass cell when the quantum-based method was used to measure the distribution of microwave magnetic field strength.

**Figure 9:**
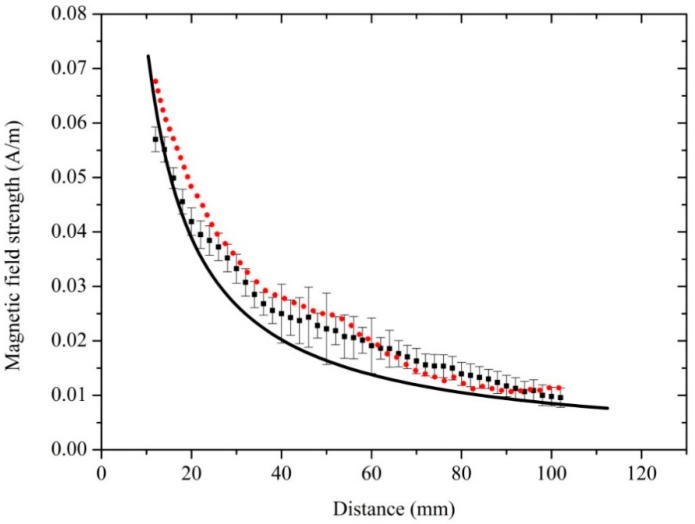
Verification results of distribution of microwave magnetic field strength. Dot line represents the simulated results of the distribution of the microwave magnetic field strength when the glass cell was inserted. The error bars on square solid spot represents the measurement uncertainty. The dot line was composed by a series of values, calculated by a series of simulation models. The frequency of electromagnetic wave was set at 9.19 GHz. We set the diameter of the column basal plane of glass cell to 20 mm, its height to 10 mm, and its thickness to 0.5 mm. The measurement distance was 52 mm. The magnetic field strengths were measured by changing the position of the Cs glass cell along the direction of o-x (showed in Figure 5c). The measurement distance was changed from 12 to 102 mm. The square solid spot represents the measured magnetic field strength, and the solid line represents the simulated field strength without the glass cell. They are consistent with the results showed in Figure 6. The variation trends of the three results were roughly identical. The simulated results of the microwave magnetic field strength when the glass cell was inserted are a little more than the simulated results in the free space at every measurement points. The measured results are much more approached to simulated results represented by the dot line.

It is necessary to accurately illustrate the influence of the electromagnetic perturbation caused by the Cs glass cell on the distribution trend of the electromagnetic field in the range of measurement distance from 12 to 102 mm. Therefore, a verification simulation of the magnetic field strength was performed to confirm the reliability of the measurement result when the glass cell was inserted. The magnetic field strength at the center of the glass cell was calculated when the measurement distance was changed from 12 to 102 mm. The calculated magnetic field strength at a relative position is shown in Figure 9. 

To facilitate comparison with the experimental results, the measurement and simulation results without the glass cell were inserted into Figure 9. The square solid spot represents the distribution of microwave magnetic field strength measured by the quantum-based sensor, the solid line the distribution of field strength simulated without the glass cell, and the circular solid spot the simulated distribution of microwave magnetic field strength when the glass cell was inserted. The microwave magnetic field strength was calculated at the center of the glass cell and the center position of the glass cell changed with measurement distance. 

As Figure 9 shows, the distribution of microwave magnetic field strength was slightly perturbed by the glass cell. A series of simulating data was obtained by changing the position of glass cell in the simulation model. The simulated magnetic field strength at the center of the glass cell changed with measurement distance from 12 to 102 mm, and was slightly higher than the magnetic field strength simulated without the glass cell. The measured results was closer to the simulated results with the glass cell in the range of 12 to 25 mm of the measurement distance, and the measured results was closer to the simulated results without the glass cell in the range of 25 to 102 mm of measurement distance. However, the distribution trend of magnetic field strength is hardly affected by the glass cell. Moreover, the distribution of magnetic field measured by the quantum-based method is consistent with that simulated with and without the glass cell. 

In our experiment, the two column basal planes of the cylindrical glass cell were not in the standard parallel configuration. The laser beam was slightly divergent and the detection of the transmission laser was affected. Furthermore, the experiment was not conducted in an anechoic chamber and the geomagnetic field was not shielded. The quantum-based measurement could be affected by the reflected microwave when the microwave power was adjusted to be too large. Moreover, the measurement could also be affected by the geomagnetic field. In addition, the laser spot was not the standard Gaussian laser spot, and the interaction between the laser and the Cs atoms could be affected by it. More accurate measurement results could be obtained when the experimental conditions mentioned above are improved.

Compared with the electron component used in the conventional measurement methods based on Maxwell formula, the atom-based sensor is more sensitive to electromagnetic fields. The experimental and simulation results demonstrate the reliability of the proposed precision electromagnetic field measurement based on atomic and molecular physics. Considering the theory and experimental process, the quantum-based method may be used in the precision measurement of the RF electromagnetic field; of the performance of RF circuits, microwave cavities, and RF waveguides; and of the concentration of the buffer gas enclosed in the alkali metal vapor cell. 

It should be pointed out that a phase modulation must be added to the electromagnetic field when the magnetic field sensor was used. However, it was unrealizable when an electromagnetic field of unknown origin wants to be measured. It was still a problem that needs to be solved. Our future research work was to measure the electromagnetic field with higher power and to extend measurement range of the quantum-based sensor. 

## 5. Conclusions

In this paper, we described a new microwave magnetic field sensor that is based on the atomic candle. The amplitude of second harmonic oscillation was enhanced when the Rabi frequency determined by the magnetic field strength was twice the frequency of the phase modulation added to the RF electromagnetic field. Cs atoms enclosed in a glass cell were used as the antenna to interact with microwaves radiated from the waveguide and the laser locked in the transition line of Cs atoms at the same time. The distribution of the magnetic field in the free space determined by the microwave power was measured by the sensor. The distribution of the magnetic field measured by the sensor and the results of the simulation calculation were in agreement. Furthermore, the electromagnetic disturbance caused by the glass cell was analyzed using simulation calculation and an electromagnetic resonance in the glass cell was found to have an effect on the magnetic field. The feasibility of the proposed quantum-based microwave magnetic field sensor was demonstrated and will be beneficial for the development of electromagnetic field precision measurement based on atomic and molecular physics.

## References

[1] Anikeev V.N. (1999). Calculation of calorimetric effect of thermo-field electron emission. IEEE Trans. Dielectr. Electr. Insul..

[2] Judaschke R., Ruhaak J. (2009). Determination of the correction factor of waveguide microcalorimeters in the millimeter-wave range. IEEE Trans. Instrum. Meas..

[3] Vremera E.T., Brunetti L., Oberto L., Sellone M. (2011). Power sensor calibration by implementing true-twin microcalorimeter. IEEE Trans. Instrum. Meas..

[4] Lee J.G., Kim J.H., Kang T.W., Won S.H., Lee D.J. (2011). RF peak power calibration of modulated signals. IEEE Trans. Instrum. Meas..

[5] Camparo J.C. (1998). Atomic stabilization of electromagnetic field strength using Rabi resonances. Phys. Rev. Lett..

[6] Sun F.Y., Ma J., Bai Q.S., Huang X.H., Gao B., Hou D. (2017). Measuring microwave cavity response using atomic Rabi resonances. Appl. Phys. Lett..

[7] Kinoshita M., Shimaoka K., Komiyama K. (2009). Determination of the microwave field strength using the Rabi oscillation for a new microwave power standard. IEEE Trans. Instrum. Meas..

[8] Kinoshita M., Shimaoka K., Komiyama K. (2011). Atomic microwave power standard based on the Rabi frequency. IEEE Trans. Instrum. Meas..

[9] Kinoshita M., Shimaoka K., Shimada Y. (2013). Optimization of the atomic candle signal for the precise measurement of microwave power. IEEE Trans. Instrum. Meas..

[10] Cetintas M., Hamid R., Sen O., Cakir S. (2010). Characterization of a far-field microwave magnetic field strength sensor based on double radiooptical resonance. IEEE Trans. Electromagn. C.

[11] Coffer J.G., Sickmiller B., Presser A., Camparo J.C. (2002). Line shapes of atomic-candle-type resonances. Phys. Rev. A.

[12] Coffer J.G., Camparo J.C. (2000). Atomic stabilization of field intensity using Rabi resonances. Phys. Rev. A.

[13] Crowley T.P., Donley E.A., Heavner T.P. (2004). Quantum-based microwave power measurements: Proof-of-concept experiment. Rev. Sci. Instrum..

[14] Sedlacek J.A., Schwettmann A., Kübler H., Löw R., Pfau T., Shaffer J.P. (2012). Microwave electrometry with Rydberg atoms in a vapour cell using bright atomic resonances. Nat. Phys..

[15] Sedlacek J.A., Schwettmann A., Kübler H., Shaffer J.P. (2013). Atom-based vector microwave electrometry using Rubidium Rydberg atoms in a vapor cell. Phys. Rev. Lett..

[16] Fan H.Q., Kumar S., Sheng J.T., Shaffer J.P., Holloway C.L., Gordon J.A. (2015). Effect of vapor-cell geometry on Rydberg-atom-based measurements of radio-frequency electric fields. Phys. Rev. Appl..

[17] Holloway C.L., Simons M.T., Gordon J.A., Wilson P.F., Cooke C.M., Anderson D.A., Raithel G. (2017). Atom-Based RF Electric Field Metrology: From Self-Calibrated Measurements to Subwavelength and Near-Field Imaging. IEEE Trans. Electromagn. C.

[18] Holloway C.L., Simons M.T., Gordon J.A., Dienstfrey A., Anderson D.A., Raithel G. (2017). Electric field metrology for SI traceability: Systematic measurement uncertainties in electromagnetically induced transparency in atomic vapor. J. Appl. Phys..

[19] Meyer D.H., Cox K.C., Fatemi F.K., Kunz P.D. (2018). Digital communication with Rydberg atoms and amplitude-modulated microwave fields. Appl. Phys. Lett..

[20] Simons M.T., Gordon J.A., Holloway C.L. (2018). Fiber-coupled vapor cell for a portable Rydberg atom-based radio frequency electric field sensor. Appl. Opt..

[21] Fan H.Q., Kumar S., Sedlacek J., Kübler H., Karimkashi S., Shaffer J.P. (2015). Atom based RF electric field sensing. J. Phys. B At. Mol. Opt. Phys..

[22] Holloway C.L., Gordon J.A., Jefferts S., Schwarzkopf A., Anderson D.A., Miller S.A., Thaicharoen N., Raithel G. (2014). Broadband Rydberg atom-based electric-field probe for SI-traceable, self-calibrated measurements. IEEE Trans. Antennas Propag..

[23] Horsley A., Treutlein P. (2016). Frequency-tunable microwave field detection in an atomic vapor cell. Appl. Phys. Lett..

[24] Horsley A., Du G.X., Treutlein P. (2015). Widefield Microwave imaging in alkali vapor cells with sub-100μm resolution. New J. Phys..

[25] Bonato C., Berry D.W. (2017). Adaptive tracking of a time-varying field with a quantum sensor. Phys. Rev. A.

[26] Yang B., Du G.X., Dong Y., Liu G.Q., Hu Z.Z., Wang Y.J. (2018). Non-invasive imaging method of microwave near field based on solid state quantum sensing. IEEE Trans. Microw. Theory.

[27] Lee S.K., Sauer K.L., Seltzer S.J., Alem O., Romalis M.V. (2006). Subfemtotesla radio-frequency atomic magnetometer for detection of nuclear quadrupole resonance. Appl. Phys. Lett..

[28] Savukov I.M., Seltzer S.J., Romalis M.V., Sauer K.L. (2005). Tunable Atomic Magnetometer for Detection of Radio-Frequency Magnetic Fields. Phys. Rev. Lett..

[29] Sun F.Y., Hou D., Bai Q.S., Huang X.H. (2018). Rabi resonance in Cs atoms and its application to microwave magnetic field measurement. J. Phys. Commun..

[30] Liu X.C., Jiang Z.Y., Qu J.F., Hou D., Huang X.H., Sun F.Y. (2018). Microwave magnetic field detection based on Cs vapor cell in free space. Rev. Sci. Instrum..

[31] Terraciano M.L., Bashkansky M., Fatemi F.K. (2008). A single-shot imaging magnetometer using cold atoms. Opt. Express.

[32] Fatemi F.K., Bashkansky M. (2010). Spatially resolved magnetometry using cold atoms in dark optical tweezers. Opt. Express.

[33] Happer W. (1972). Optical pumping. Rev. Mod. Phys..

[34] Demtröder W. (2015). Laser Spectroscopy 2.

[35] Kroemer E., Abdel H.M., Maurice V., Fouilland B., Gorecki C., Boudot R. (2015). Cs vapor microcells with Ne-He buffer gas mixture for high operation-temperature miniature atomic clocks. Opt. Express.

[36] Beverini N., Strumia F. (1981). Buffer gas pressure shift in the m_F_=0→m_F_=0 ground state hyperfine line in Cs. Opt. Commun..

